# Seipin deficiency in mice causes loss of dopaminergic neurons via aggregation and phosphorylation of α-synuclein and neuroinflammation

**DOI:** 10.1038/s41419-018-0471-7

**Published:** 2018-04-18

**Authors:** Ling Wang, Juan Hong, Yajuan Wu, George Liu, Wenfeng Yu, Ling Chen

**Affiliations:** 1 State Key Lab of Reproductive Medicine, Nanjing Medical University, 210029 Nanjing, China; 2 Department of Physiology, Nanjing Medical University, 210029 Nanjing, China; 3 Institute of Cardiovascular Sciences, Peking University and Key Laboratory of Cardiovascular Sciences, China Administration of Education, 100191 Beijing, China; 4 Key Lab of Molecular Biology, Guiyang Medical University, 550004 Guiyang, Guizhou China

## Abstract

Seipin gene is originally found in type 2 congenital generalized lipodystrophy (CGL2) to involve lipid droplet formation. Recently, decrease of seipin expression is reported in substantia nigra of Parkinson’s disease patients. Dopaminergic neurons in substantia nigra pars compacta expressed the seipin protein. The objective of this study is to investigate influence of the seipin deficiency on dopaminergic neurons and motor behaviors. Neuronal *seipin* knockout (*seipin*-nKO) mice (3–12 months of age) displayed an age-related deficit in motor coordination. The number of dopaminergic neurons in *seipin*-nKO mice was age dependently reduced with increase in cleaved caspase-3. The levels of αSyn oligomers and oligomer phosphorylation (S129), but not αSyn monomers, were elevated in dopaminergic neurons and substantia nigra of *seipin*-nKO mice. The PPARγ expression in *seipin*-nKO mice was reduced. In *seipin*-nKO mice, the phosphorylation of GSK3β was increased at Tyr216 and was reduced at Ser9, which was corrected by the PPARγ agonist rosiglitazone. The increased IL-6 level in *seipin*-nKO mice was sensitive to rosiglitazone and GSK3β inhibitor AR-A014418. The enhanced phosphorylation of αSyn was prevented by rosiglitazone and AR-A014418, while the increase in αSyn oligomers was corrected only by rosiglitazone. The treatment of *seipin*-nKO mice with rosiglitazone and AR-A014418 rescued the death of dopaminergic neurons, which was accompanied by the improvement of motor coordination. Therefore, the results indicate that seipin deficiency causes an age-related loss of dopaminergic neurons and impairment of motor coordination through reducing PPARγ to enhance aggregation and phosphorylation of αSyn and neuroinflammation.

## Introduction

Congenital generalized lipodystrophy (CGL) is an autosomal recessive disorder characterized by a near-total loss of adipose tissue, severe insulin resistance, hypertriglyceridemia, and fatty liver^1,2^. Patients with CGL2 have sensorineural deafness and cognitive disorder^3^ by mutation in the Berardinelli-Seip congenital lipodystrophy 2 gene that encodes seipin^4,5^. Seipin has been reported in various neurological syndromes^6^. Heterozygosity for missense mutations (N88S/S90L) in seipin is associated with a broad spectrum of motoneuron diseases, such as seipinopathy^7^. The motor neurons and peripheral motor axons are differentially affected in patients with seipin mutations^8,9^. The overexpression of human seipin mutants in mice caused the death of alpha motor neurons in the spinal cord which was not associated with total loss of adipose tissue^10^. In contrast, the loss-of-function mutations in seipin gene did not cause the death of alpha motor neurons in mice; however, neuronal *seipin* knockout (*seipin*-nKO) mice at 2 months of age have a tendency to decrease the latency of falling off during the rotarod test (RT)^11^.

The proteomic or Western blotting analysis of human demonstrated a 2.5-fold decrease of seipin expression levels in the substantia nigra (SN) of Parkinson’s disease patients^12^. The protein was observed similarly diminished in the locus coeruleus of Parkinson’s disease patients, another aminergic nucleus affected earlier than SN in the disease process^13^. Parkinson’s disease is a common neurodegenerative disorder characterized by the gradual dysfunction of the extrapyramidal motor system and progressive loss of dopaminergic neurons in substantia nigra pars compacta (SNpc). Susceptible nerve cells exhibit intracellular inclusions termed Lewy bodies formed by the abnormal accumulation and aggregation of α-synuclein (αSyn)^14^. The accumulation of oligomeric αSyn may cause the degeneration of dopaminergic neurons. Seipin, an endoplasmic reticulum (ER)-resident membrane protein, plays a role in the generation of peroxisome proliferator-activated receptor gamma (PPARγ). Seipin deficiency via reducing PPARγ increases glycogen synthase kinase-3β (GSK-3β) activity, which increases in the levels of interleukin-6 (IL-6) and tumor necrosis factor-α (TNF-α)^15^. The activation of GSK-3β promotes inflammatory responses in the brain^16^. In brain of Parkinson’s disease, a glial reaction increases the release of various inflammatory factors. The activation of PPARγ or the inhibition of GSK-3β has been reported to decrease αSyn protein expression and oligomerization^17,18^. In addition, the *seipin* knockdown is known to cause the accumulation of phosphatidic acids in yeast^2^, adipose cells^19^, and neuronal cells^20^. The exposure of cultured dopaminergic neurons to physiological polyunsaturated fatty acid concentrations increases the levels of soluble αSyn oligomers and insoluble αSyn aggregates and enhances its deposition into cytoplasmic intraneuronal Lewy-like inclusions^21^. Therefore, it is of great interest to investigate whether the seipin deficiency causes Parkinson’s disease pathogenesis.

## Results

### Influence of seipin deficiency on motor coordination

**Fig. 1:**
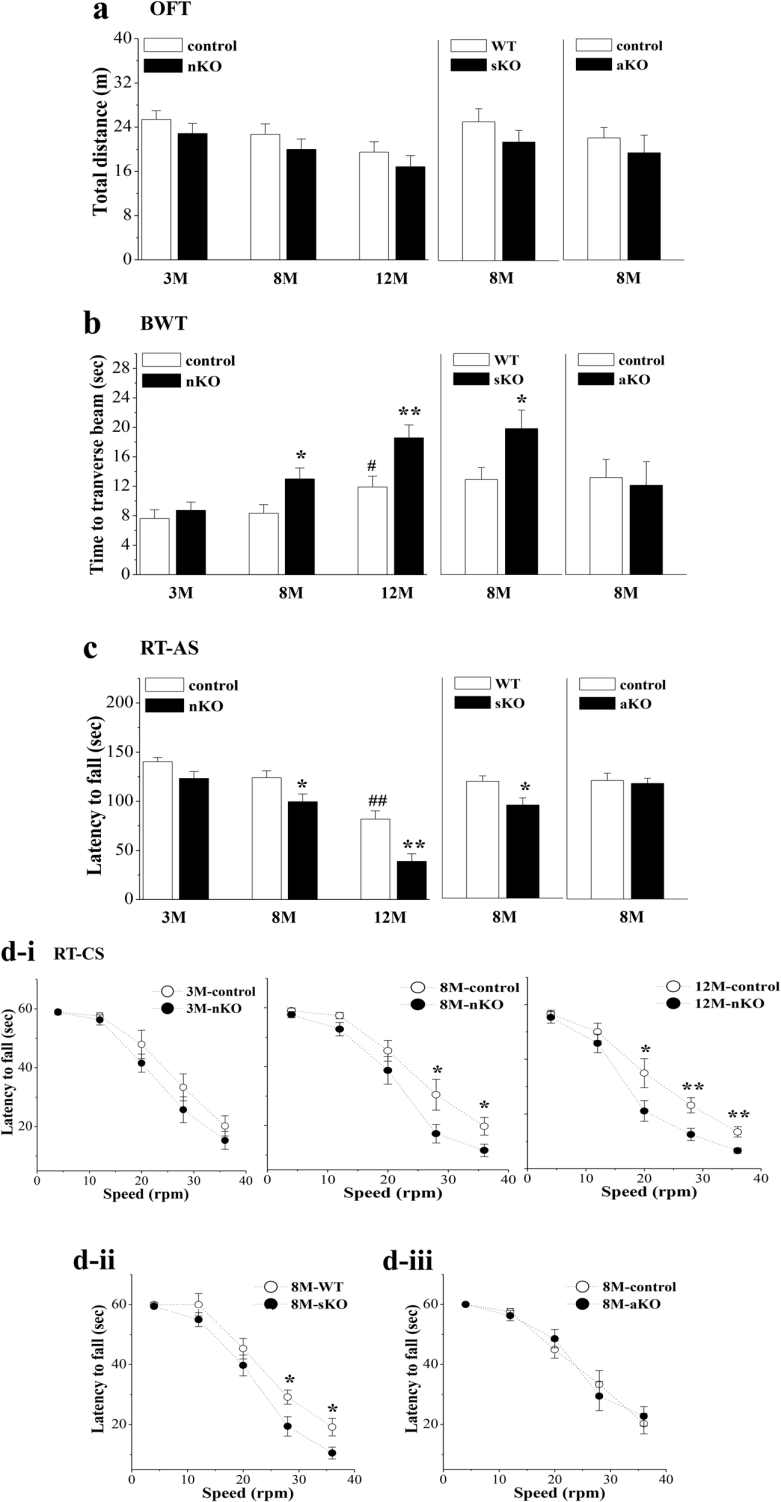
Neuronal seipin deficiency impairs motor coordination. **a** Influence of neuronal seipin deficiency on spontaneous locomotion as assessed by OFT. Bar graph shows traveled distance in 3-M-old (3 M), 8-M-old (8 M), and 12-M-old (12 M) control and *seipin*-nKO mice; 8-M-old WT and *seipin*-sKO mice; and 8-M-old control and *seipin*-aKO mice. **b** Bar graph shows time (s) to traverse the beam in BWT. *P < 0.05 and **P < 0.01 vs. age-matched control mice (two-way ANOVA) or WT mice (*t-*test). #*P* < 0.05 vs. 3-M-old control mice (two-way ANOVA). **c** Each point represents the latency (s) on rotated rod with accelerating speed (RT-AS). *P < 0.05 and **P < 0.01 vs. age-matched control mice (two-way ANOVA) or WT mice (*t*-test). ^##^*P* < 0.01 vs. 3-M-old control mice (two-way ANOVA). **d** Bars indicate the latency (s) on rotated rod with constant speeds (RT-CS) in control mice and *seipin*-nKO mice (**d**-i), 8-M-old WT mice and *seipin*-sKO mice (**d**-ii), and 8-M-old control mice and *seipin*-aKO mice (**d**-iii). *P < 0.05 and **P < 0.01 vs. age-matched control mice or WT mice (repeated measures ANOVA)

Spontaneous activity was initially examined using an open-field test (OFT). The group means for the total distance traveled in 3-month-old (M-old), 8-M-old, and 12-M-old *seipin*-nKO mice, 8-M-old *seipin*-sKO mice or 8-M-old *seipin*-aKO mice did not significantly differ from those of age-matched control mice (nestin-Cre mice) or wild-type (WT) mice (*P* > 0.05, *n* = 12; Fig. 1a). The beam walking test (BWT) (Fig. 1b) and RTs with accelerating speed (RT-AS; Fig. 1c) or constant speed (RT-CS; Fig. 1d) were used to estimate the ability of motor coordination. In *seipin*-nKO mice, the time that mice traversed the beam in BWT or the latency that mice remained on the rotarod before falling off were affected by genotype (BWT: *F*_(1, 66)_ = 13.472, *P* < 0.001; RT-AS: *F*_(1, 66)_ = 23.029, *P* < 0.001; RT-CS: *F*_(1, 66)_ = 20.707, *P* < 0.001). Compared to control mice, 8-M-old and 12-M-old *seipin*-nKO mice spent a longer time traversing the beam in the BWT (8M: *P* < 0.05; 12M: *P* < 0.01) and remained on the rotarod for less time in the RT-CS (8M: 28 rpm: *P* < 0.05, 36 rpm: *P* < 0.05; 12M: 20 rpm: *P* < 0.05, 28 or 36 rpm: *P* < 0.01) or RT-AS (8M: *P* < 0.05; 12M: *P* < 0.01). Although 3-M-old *seipin*-nKO mice had a tendency to have an increased walking time in the BWT and a decreased latency in the RT-CS or RT-AS, the group comparison with control mice failed to reach significance (*P* > 0.05). Consistently, 8-M-old *seipin*-sKO mice required more time to traverse the beam in the BWT (*P* < 0.05, *n* = 12) and had a decreased latency on the rotarod of RT-CS (*P* < 0.05, *n* = 12) or RT-AS (*P* < 0.05, *n* = 12) than WT mice. By contrast, the time spent to traverse the beam in the BWT or remain on the rotarod in the RT-CS and RT-AS was not significantly different between 8-M-old control mice (nestin-Cre mice) and *seipin*-aKO mice (*P* > 0.05, *n* = 12). These results indicate that neuronal seipin deficiency causes an age-related progressive decline in motor coordination.

### Influence of seipin deficiency on dopaminergic neurons in the SNpc

**Fig. 2:**
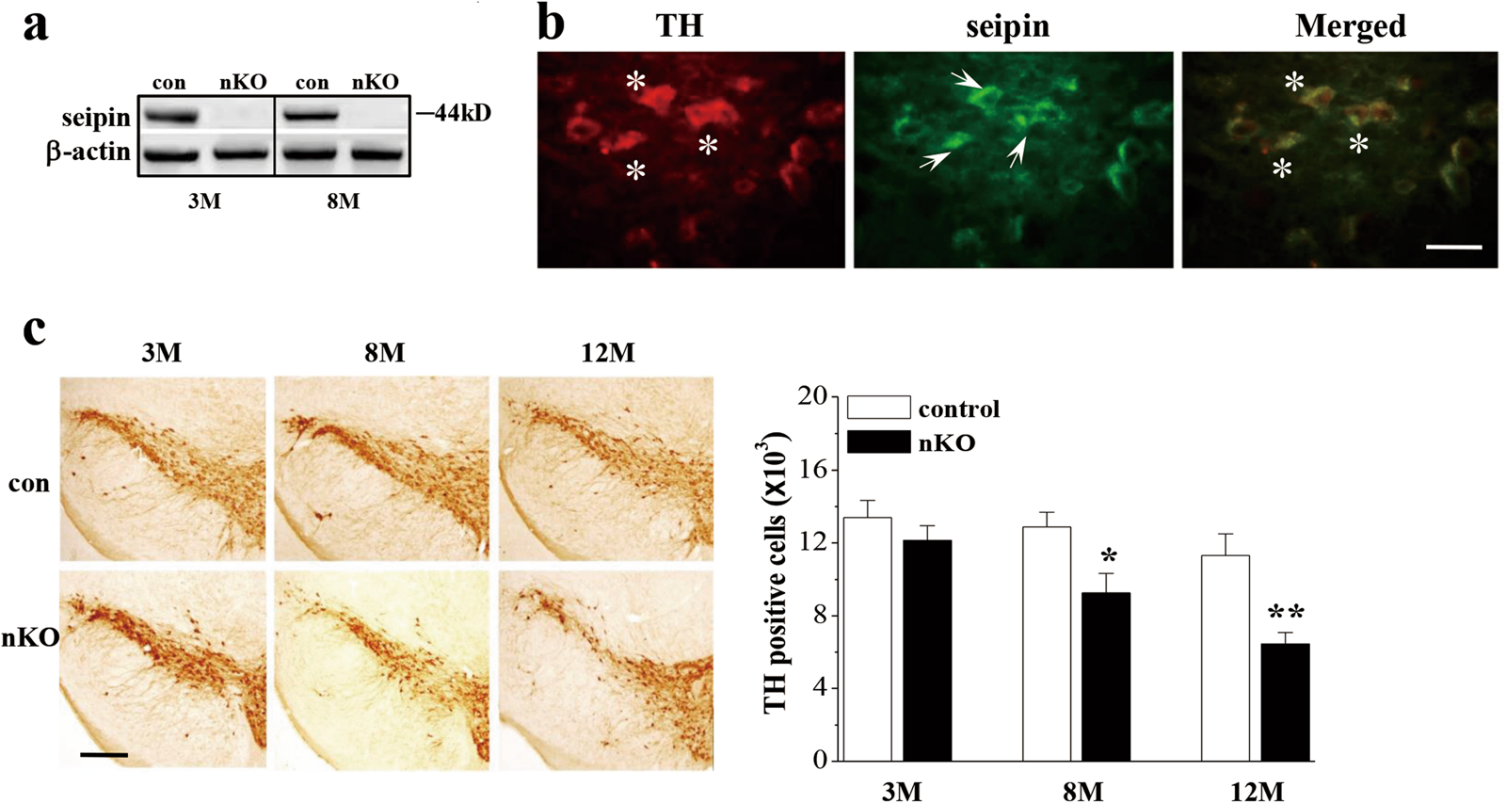
Neuronal seipin deficiency causes a loss of dopaminergic neurons in the SNpc. **a** Levels of seipin protein in SN of 3-M-old (3 M) and 8-M-old (8 M) control mice (con) and *seipin*-nKO mice (nKO). **b** Representative fluorescence images of seipin (green) and tyrosine hydroxylase (TH, red) double immuno-staining in SNpc of 8-M-old control mice (scale bar = 50 μm). The arrows indicate seipin-positive cells; the asterisks represent TH-positive cells. **c** Stereological counts of TH-positive cells throughout SNpc of 3-M-old (3 M), 8-M-old (8 M), and 12-M-old (12 M) control mice and *seipin*-nKO mice. *P < 0.05 and **P < 0.01 vs. age-matched control mice (two-way ANOVA). Representative photographs of TH staining. Scale bar = 200 μm

The western blotting analysis showed the expression of seipin protein at approximately 44 kD in the SN of 3-M-old and 8-M-old control mice (Fig. 2a) and a lack of seipin protein in *seipin*-nKO mice. Using seipin and tyrosine hydroxylase (TH) double immunohistochemical staining, we observed the seipin-positive dopaminergic neurons in SNpc of 8-M-old control mice (Fig. 2b).

To explore the cellular mechanisms underlying impaired motor coordination caused by seipin deficiency, we performed a stereological count of TH-positive cells. The number of TH-positive cells was affected by genotype (*F*_(1, 30)_ = 18.480, *P* < 0.001; Fig. 2c). In comparison with control mice, the number of TH-positive cells was reduced by approximately 9% in 3-M-old *seipin*-nKO mice (*P* > 0.05), 28% in 8-M-old *seipin*-nKO mice (*P* < 0.05), and 43% in 12-M-old *seipin*-nKO mice (*P* < 0.01). These results indicate that seipin deficiency causes an age-related progressive loss of dopaminergic neurons.

### Influence of seipin deficiency on oligomerization and phosphorylation of αSyn in SN

**Fig. 3:**
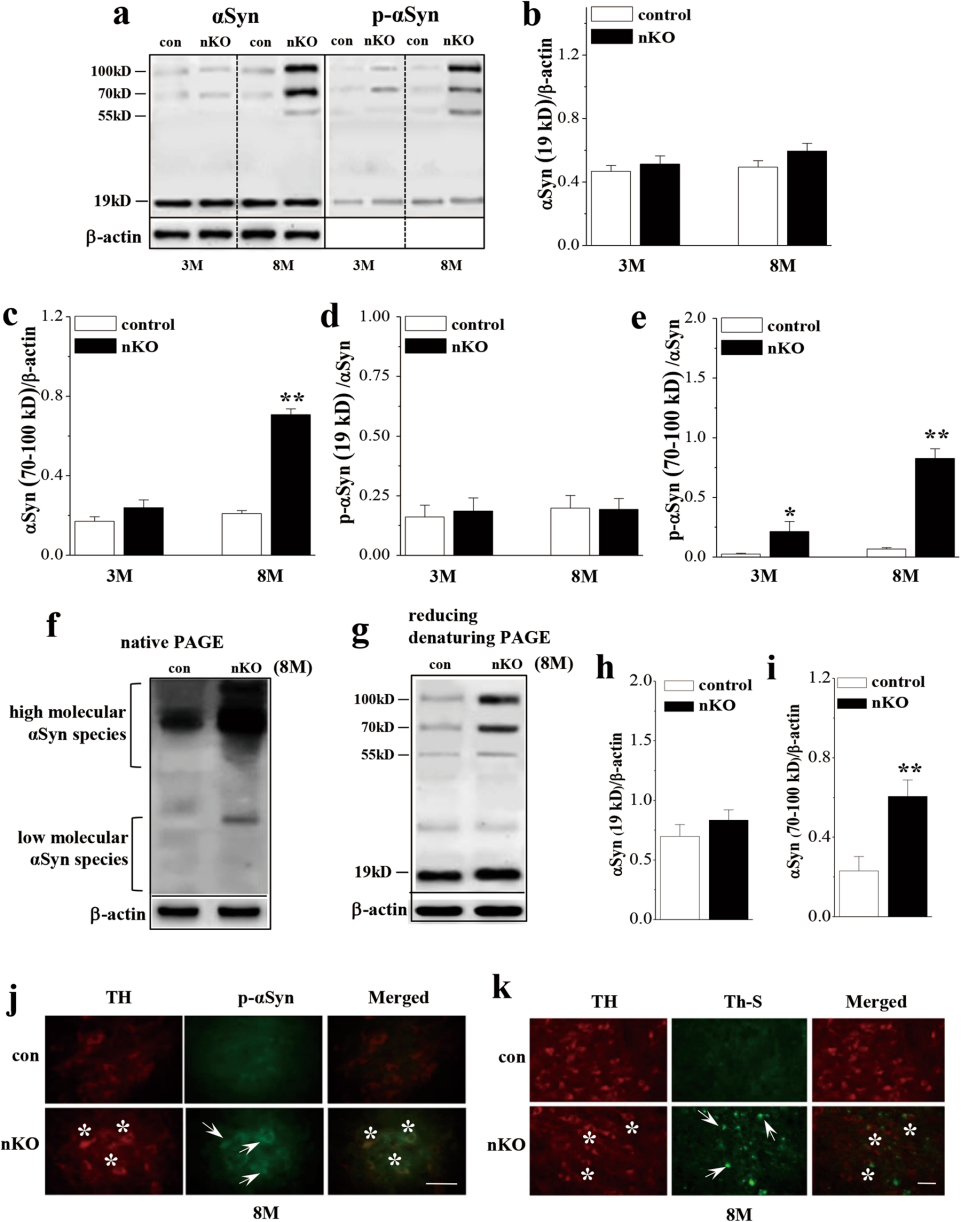
Neuronal seipin deficiency increases the accumulation or phosphorylation of αSyn. **a** Representative immunoblots of αSyn and phosphor-αSyn in SN of 3-M-old and 8-M-old control and *seipin*-nKO mice. The data of β-actin in αSyn and phosphor-αSyn are the same. **b**–**e** The densitometric values for monomers (19 kD) and oligomers of total and phosphorylation of αSyn (p-αSyn). *P < 0.05 and **P < 0.01 vs. control mice (two-way ANOVA). **f** Representative immunoblots of αSyn in a native PAGE in 8-M-old control (con) and *seipin*-nKO (nKO) mice. **g**–**i** Bar graphs show the densitometric values of αSyn monomers and oligomers using reducing denaturing PAGE. **P < 0.01 vs. control mice (*t*-test). **j**, **k** Double immuno-staining photographs of phosphor-αSyn (p-αSyn, green) and TH (red) or Th S (green) and TH (red) in SNpc of 8-M-old control and *seipin*-nKO mice. The arrows indicate p-αSyn or Th S-positive cells; the asterisks represent TH-positive cells. Scale bars = 50 μm (**j**); scale bars = 50 μm (**k**)

To investigate the molecular mechanisms underlying the loss of dopaminergic neurons by seipin deficiency, the oligomerization and phosphorylation (S129) of αSyn were examined using non-reducing denaturing PAGE in the SN of 3-M-old and 8-M-old control mice and *seipin*-nKO mice (Fig. 3a). The levels of αSyn oligomers were affected by genotype (*F*_(1, 20)_ = 101.019, *P* < 0.001; Fig. 3c) and genotype × age (*F*_(1, 20)_ = 57.588, *P* < 0.001). The level of αSyn oligomers (70–100 kD) was higher in 8-M-old *seipin*-nKO mice than that in age-matched control mice (*P* < 0.01). Although 3-M-old *seipin*-nKO mice had a tendency to increase the oligomerization of αSyn, the group comparison with control mice failed to reach significance (*P* > 0.05). Consistently, the increase of αSyn oligomers in 8-M-old seipin-nKO mice was determined by the examination of native PAGE (Fig. 3f). The analysis of reducing denaturing PAGE in seipin-nKO mice (Fig. 3g) showed a similar increase in the level of αSyn oligomers (*P* < 0.01, *n* = 6; Fig. 3i). In addition, the phosphorylation of αSyn oligomers (p-αSyn) was affected by genotype (*F*_(1, 20)_ = 65.58, *P* < 0.001; Fig. 3e) and genotype × age (*F*_(1, 20)_ = 23.769, *P* < 0.001). The level of αSyn phosphorylation (ratio of p-αSyn/total αSyn oligomers) in 3-M-old (*P* < 0.05) or 8-M-old *seipin*-nKO mice (*P* < 0.01) was elevated compared to that in control mice. In contrast, neither the levels of the αSyn monomer (19 kD) (*P* > 0.05, *n* = 6; Fig. 3b) nor the levels of phosphorylated αSyn monomer (*P* > 0.05, *n* = 6; Fig. 3d) in *seipin*-nKO mice were different from those of control mice. The αSyn monomer by reducing denaturing PAGE showed a slight increase in *seipin*-nKO mice, but the group when compared with control mice failed to reach the significance (*P* > 0.05, *n* = 6; Fig. 3h).

The double-immunofluorescence staining of αSyn phosphorylation (p-αSyn) and TH showed the elevation of the phosphorylated αSyn in TH-positive cells of 8-M-old *seipin*-nKO mice (Fig. 3j). By double-immunofluorescence staining of TH and thioflavin S, a dye that binds to amyloid fibrils^22^, we observed that the TH-positive cells in the 8-M-old *seipin*-nKO mice was highly overlapped by the thioflavin S-positive structures (Fig. 3k), whereas the thioflavin S-positive structures were barely found in control mice. These results indicate that the seipin deficiency enhances an age-related accumulation and phosphorylation of αSyn, and αSyn fibril formation in dopaminergic neurons.

### Influence of seipin deficiency reduced PPARγ on neuroinflammation in SN

**Fig. 4:**
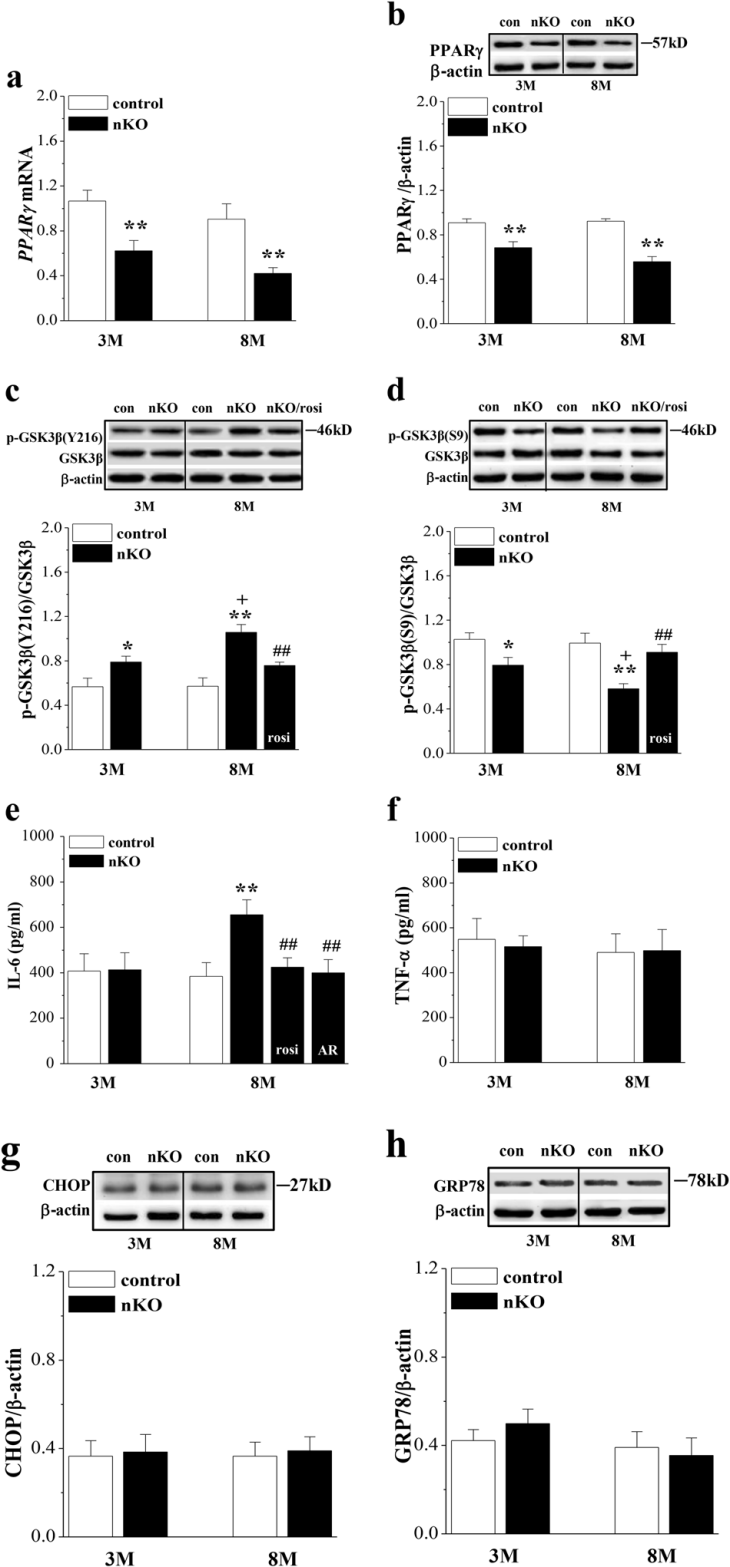
Neuronal seipin deficiency increases GSK3β activity and IL-6 production. **a**, **b** The bars represent the levels of *PPARγ* mRNA and PPARγ protein in SN of 3-M-old and 8-M-old control and *seipin*-nKO mice. **P < 0.01 vs. control mice (two-way ANOVA). **c**, **d** The densitometric values for GSK3β phosphorylation (p-GSK3β) at Ser9 and Tyr216 in SN of 3-M-old and 8-M-old control and *seipin*-nKO mice treated with rosiglitazone (rosi) for 7 days. *P < 0.05 and **P < 0.01 vs. control mice; +*P* < 0.05 vs. 3-M-old *seipin*-nKO mice; ##*P* < 0.01 vs. 8-M-old *seipin*-nKO mice (three-way ANOVA). **e**, **f** Levels of IL-6 and TNF-α in SN of 3-M-old and 8-M-old control and *seipin*-nKO mice treated with rosi or the GSK3β inhibitor AR-A014418 (AR) for 7 days. **P < 0.01 vs. control mice; #*P* < 0.05 and ##*P* < 0.01 vs. 8-M-old *seipin*-nKO mice (three-way ANOVA). **g**, **h** Levels of CHOP and GRP78 protein in SN of 3-M-old and 8-M-old control and *seipin*-nKO mice

Consistent with the previous reports^11,20^, the levels of *PPARγ* mRNA (*P* < 0.01, *n* = 6; Fig. 4a) and PPARγ protein (*P* < 0.01, *n* = 6; Fig. 4b) in the SN of 3-M-old and 8-M-old *seipin*-nKO mice were reduced compared to control mice.

Seipin deficiency in astrocytes has been reported to increase GSK3β activity and levels of pro-inflammatory cytokines through reducing PPARγ^15^. GSK3β activity is negatively and positively regulated by phosphorylation (phospho-GSK3β) at Ser9 and Tyr216, respectively. There was a main effect of seipin deficiency on phospho-GSK3β at Tyr216 (*F*_(1, 20)_ = 25.970, *P* < 0.001; Fig. 4c) and Ser9 (*F*_(1, 20)_ = 22.151, *P* < 0.001; Fig. 4d). The level of Tyr216 phospho-GSK3β was elevated (3 M: *P* < 0.05; 8 M: *P* < 0.01), and the level of Ser9 phospho-GSK3β was reduced (3 M: *P* < 0.05; 8 M: *P* < 0.01) in 3-M-old and 8-M-old *seipin*-nKO mice compared to those in the controls. Notably, the level of Tyr216 phospho-GSK3β showed an age-related increase in *seipin*-nKO mice (*P* < 0.05). Importantly, the treatment of 8-M-old *seipin*-nKO mice with the PPARγ agonist rosiglitazone (rosi, 5 mg/kg) for 7 days obviously corrected the elevation of Tyr216 phospho-GSK3β (*P* < 0.01) and the reduction of Ser9 phospho-GSK3β (*P* < 0.01). The level of IL-6 in the SN was affected by genotype (*F*_(1, 28)_ = 29.253, *P* < 0.001; Fig. 4e) and genotype × age (*F*_(1, 28)_ = 29.253, *P* < 0.001). The elevation of IL-6 in 8-M-old *seipin*-nKO mice was sensitive to the 7 days administration of rosi (*P* < 0.01, *n* = 10) or the GSK3β inhibitor AR-A014418 (AR, 1 mg/kg, *P* < 0.01, *n* = 10). By contrast, the level of TNF-α in the SN of 3-M-old and 8-M-old *seipin*-nKO mice did not significantly differ from control mice (*P* > 0.05, *n* = 10; Fig. 4f).

Mutations in seipin can induce ER stress^6^. However, the levels of CHOP (C/EBP homologous protein, transcribed following PERK activation) (*P* > 0.05, *n* = 6; Fig. 4g) or GRP78 protein (*P* > 0.05, *n* = 6; Fig. 4h) in the SN were not significantly different between 3-M-old or 8-M-old control mice and *seipin*-nKO mice. These results indicate that seipin deficiency, through reducing PPARγ, enhances age-dependent GSK3β activation, leading to neuroinflammation.

### Involvement of αSyn in loss of dopaminergic neurons and impaired motor coordination

**Fig. 5:**
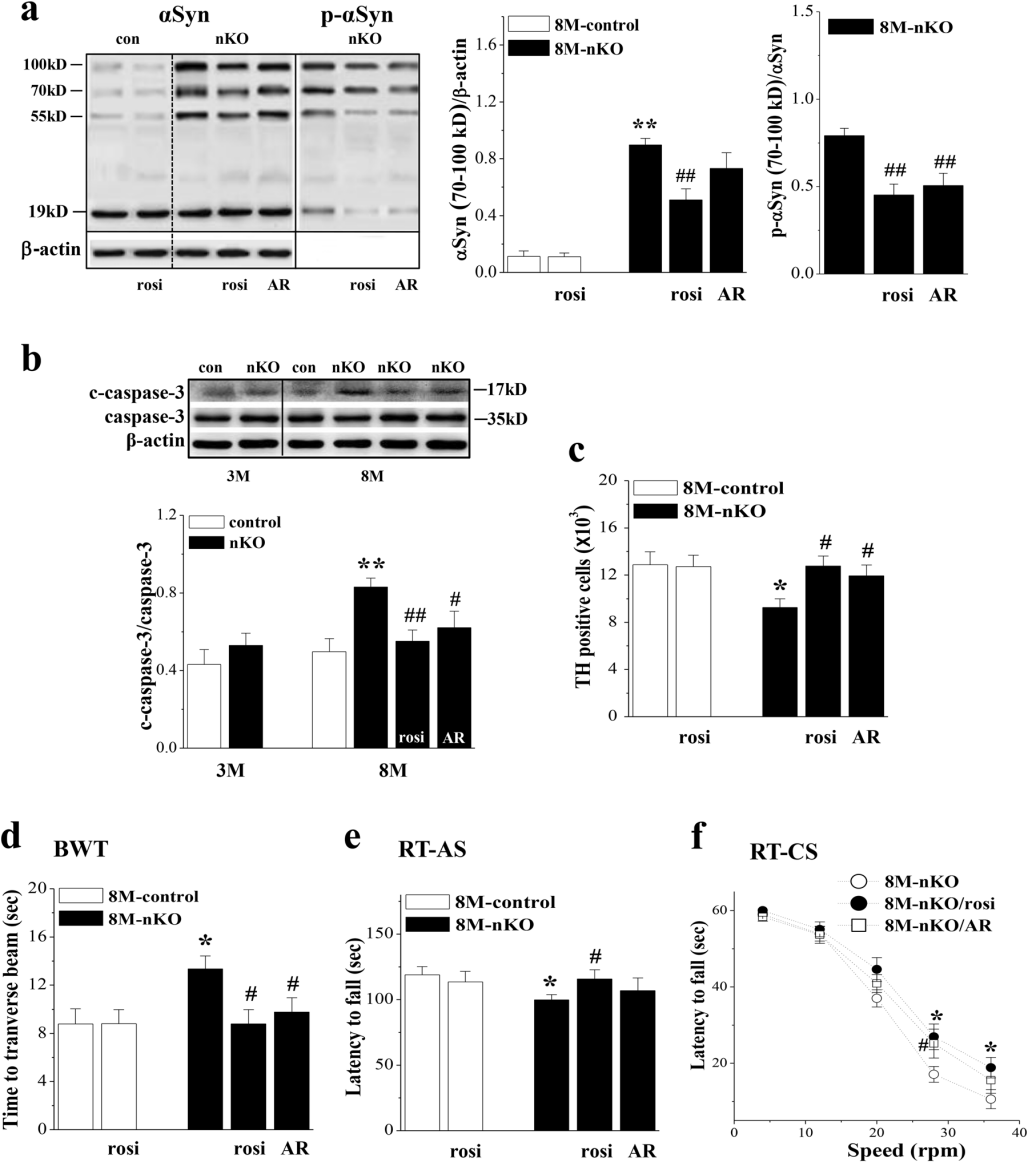
Effects of rosiglitazone and AR-A014418 on accumulation and phosphorylation of αSyn and impairment of dopaminergic neurons and motor coordination. **a** Bar graph shows the densitometric values of αSyn oligomers and phosphorylation of αSyn oligomers (p-αSyn) in SN of 8-M-old control and *seipin*-nKO mice treated with rosi or AR-A014418 (AR) for 28 days. **P < 0.01 vs. control mice; ##*P* < 0.01 vs. *seipin*-nKO mice (two-way ANOVA). The data of β-actin in αSyn oligomers and phosphorylation of αSyn oligomers are the same. **b** Level of cleaved caspase-3 (c-caspase-3) in SN of 3-M-old and 8-M-old control mice and *seipin*-nKO mice treated with rosi or AR. **P < 0.01 vs. control mice; #*P* < 0.05 and ##*P* < 0.01 vs. 8-M-old *seipin*-nKO mice (three-way ANOVA). **c** Bar graph indicates the number of TH-positive cells in SNpc of 8-M-old control mice and *seipin*-nKO mice treated with rosi or AR. *P < 0.05 vs. control mice; #*P* < 0.05 vs. *seipin*-nKO mice (two-way ANOVA). **d**, **e** Bar graphs show the time (s) to traverse the beam in BWT and the latency (s) in RT-AS. *P < 0.05 vs. control mice; #*P* < 0.05 vs. *seipin*-nKO mice (two-way ANOVA). **f** Each point represents the latency in RT-CS. *P < 0.05 (*seipin*-nKO mice treated with rosi) and #*P* < 0.05 (*seipin*-nKO mice treated with AR) vs. *seipin*-nKO mice (repeated measures ANOVA)

To determine the involvement of reduced PPARγ and elevated activity of GSK3β in the oligomerization and phosphorylation of αSyn, 7-M-old *seipin*-nKO mice were treated with rosi (5 mg/kg) or AR (1 mg/kg) for 28 days. The treatment of 8-M-old *seipin*-nKO mice with rosi could correct the increase in the levels of αSyn oligomers (*P* < 0.01, *n* = 6; Fig. 5a) and phosphorylation of αSyn (*P* < 0.01, *n* = 6). Similarly, the AR administration in 8-M-old *seipin*-nKO mice could attenuate the phosphorylation of αSyn (*P* < 0.01, *n* = 6), but it had no effect on the oligomerization of αSyn (*P* > 0.05, *n* = 6).

Finally, we examined the effects of rosi (5 mg/kg) or AR (1 mg/kg) on the loss of dopaminergic neurons and impairment of motor coordination in 8-M-old *seipin*-nKO mice. As shown in Fig. 5b, the level of cleaved caspase-3 (c-caspase-3) in the SN of 8-M-old *seipin*-nKO mice was higher than that in control mice (*P* < 0.01, *n* = 6), whereas there was no significant difference between 3-M-old control mice and *seipin*-nKO mice (*P* > 0.05, *n* = 6). The treatment of *seipin*-nKO mice with rosi perfectly prevented the increase in the level of c-caspase-3 (*P* < 0.01, *n* = 6) and the loss of dopaminergic neurons (*P* < 0.05, *n* = 6; Fig. 5c). Furthermore, the deficits in motor coordination of 8-M-old *seipin*-nKO mice were significantly relieved by the administration of rosi (BWT: *P* < 0.05, *n* = 12; Fig. 5d, RT-AS: *P* < 0.05, *n* = 12; Fig. 5e, RT-CS: *P* < 0.05, *n* = 12; Fig. 5f). The administration of rosi alone in control mice did not cause changes in motor coordination (BWT: *P* > 0.05, *n* = 12; RT-AS: *P* > 0.05, *n* = 12). In contrast, the inhibited GSK3β activation by AR partially reduced the level of c-caspase-3 (*P* < 0.05, *n* = 6) and the death of dopaminergic neurons (*P* < 0.05, *n* = 6), and could alleviate the deficits in motor coordination (BWT: *P* < 0.05, *n* = 12; RT-CS: *P* < 0.05, *n* = 12) in 8-M-old *seipin*-nKO mice.

## Discussion

Using the *seipin*-sKO mice, *seipin*-nKO mice, and *seipin*-aKO mice models, the present study provides evidence that seipin deficiency in dopaminergic neurons through reducing PPARγ expression enhances the aggregation and phosphorylation of αSyn and induces the neuroinflammatory, which leads to the loss of dopaminergic neurons and the deficits of motor coordination. This conclusion is deduced mainly from the following observations. First, 8-M-old and 12-M-old *seipin*-nKO mice and 8-M-old *seipin*-sKO mice exhibited decline in motor coordination, but the *seipin*-aKO mice did not. Therefore, it is conceivable that the deficiency in motor coordination is induced by the neuronal seipin deficiency, which is unlikely to be secondary to the peripheral metabolic abnormalities. In addition, the neuronal seipin deficiency does not affect motor ability, as the phenotypes of motor neurons impaired by the gain-of-function mutation in the N-glycosylation site of seipin^6^ are not observed in *seipin*-sKO, *seipin*-nKO, and *seipin*-aKO mice^11^ in the present study or in *seipin* knockout rats^23^. Second, the dopaminergic neurons in the SNpc highly expressed the seipin protein. The number of TH-positive cells was reduced by approximately 28 and 43% in 8-M-old and 12-M-old *seipin*-nKO mice. Third, the levels of αSyn oligomer, phosphor-αSyn, and IL-6 in the SN of 8-M-old *seipin*-nKO mice were significantly increased. Moreover, the seipin deficiency increased the αSyn fibril formation in dopaminergic neurons. Fourth, the activation of PPARγ by rosiglitazone in 8-M-old *seipin*-nKO mice could reduce the aggregation and phosphorylation of αSyn and correct the elevation of IL-6, and prevent the loss of dopaminergic neurons, which was accompanied by the recovery of motor coordination.

### Seipin deficiency enhances the oligomerization of αSyn by reducing PPARγ

The level of PPARγ in the SN of *seipin*-nKO mice was reduced, and the PPARγ activator could prevent αSyn oligomerization. It is highly likely that the reduction of PPARγ enhances the αSyn oligomerization. The idea is supported by a recent study^24^ that PPARγ, as a binding partner for transcription factor retinoic X receptor, prevents the formation of αSyn oligomers. Growing evidence suggests that polyunsaturated fatty acids can promote αSyn oligomerization and aggregation^25^. The exposure of dopaminergic neurons to physiological polyunsaturated fatty acids concentrations can elevate the levels of soluble αSyn oligomers and insoluble αSyn aggregates^26^ and increases the deposition of αSyn into cytoplasmic intraneuronal Lewy-like inclusions^21^. The seipin deficiency has been reported to induce the accumulation of phosphatidic acids in neuronal cells, which is able to suppress the PPARγ expression^20^. Thus, one possible explanation is that the seipin deficiency via the accumulation of phosphatidic acids leads to the decline of PPARγ concentration, which enhances the αSyn oligomerization. In addition, the αSyn phosphorylation, especially at serine 129, has been shown to promote αSyn aggregation^27^. The activation of GSK-3β has been reported to affect αSyn protein expression and oligomerization^17,18^. A recent study has reported that the GSK-3β inhibitors can decrease the αSyn accumulation^28^. However, the inhibition of GSK-3β by AR-A014418 failed to alter the oligomerization of αSyn in *seipin*-nKO mice, although it reduced the phosphorylation of αSyn.

### Seipin deficiency enhances αSyn phosphorylation through GSK-3β activation

Serine 129 phosphorylation is one of the most important post-translational modifications of αSyn. The phosphorylated S129 αSyn is co-localized with phosphorylated Tyr216 GSK-3β. And, the activation of GSK-3β can directly phosphorylate αSyn at a single site, S129^29^. In the SN of *seipin*-nKO mice, the catalytic activity of GSK3β was enhanced as indicated by the elevation of Tyr216 phospho-GSK3β and the reduction of Ser9 phospho-GSK3β without the changes in the level of GSK3β protein, which could be corrected by the PPARγ agonist. Accumulating evidence supports the notion that GSK3β is a strong promoter of pro-inflammatory cytokines, including IL-6 and TNF-α. The level of IL-6 was elevated in the SN of *seipin*-nKO mice, whereas the level of TNF-α was not altered significantly. TNF-α is mainly released by activated microglia. The seipin protein is expressed in astrocytes, not in microglia^15^. Thus, the seipin deficiency in astrocytes increases the production of IL-6 via the activation of GSK3β. The elevation of IL-6 and phosphorylation of αSyn in 8-M-old *seipin*-nKO mice were sensitive to the GSK3β inhibitor and the PPARγ agonist, but it remains unclear whether αSyn phosphorylation is consequence of inflammation. On the other hand, ER stress is known to promote αSyn phosphorylation^30^. The αSyn phosphorylation can induce the activation of ER stress^31^. Consistent with the results observed in the hippocampus^11^, the levels of the ER stress markers GRP78 and CHOP were not increased in the SN of *seipin*-nKO mice.

### Seipin deficiency leads to an age-related loss of dopaminergic neurons

**Fig. 6:**
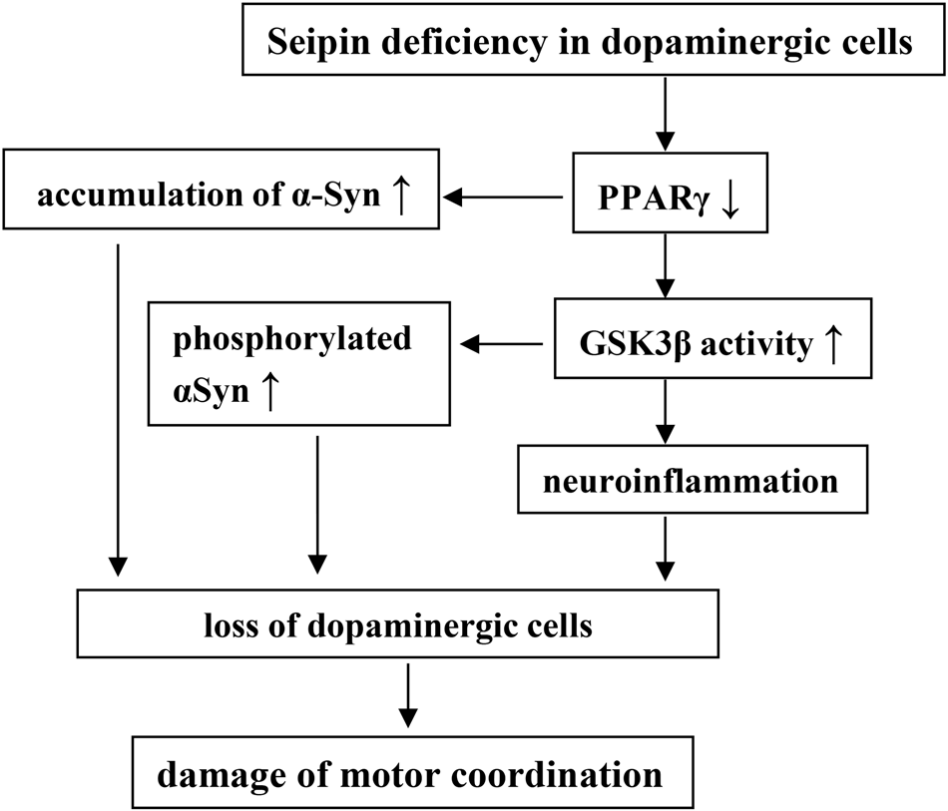
Possible mechanisms underlying seipin deficiency increased loss of dopaminergic neurons. ↑: increase; ↓: decrease

Another critical finding in the present study is that *seipin*-nKO mice showed an age-related loss of dopaminergic neurons and deficits in motor coordination. The administration of the PPARγ agonist or the GSK3β inhibitor in *seipin*-nKO mice could reduce the death of dopaminergic neurons and improve the deficits in motor coordination, although the PPARγ-mediated effects seem to be larger than those of the GSK3β inhibitor. The reduced PPARγ expression or the activated phosphorylation of GSK-3β was detected in young *seipin*-nKO mice, while the accumulation and phosphorylation of αSyn or the level of IL-6 were increased in an age-dependent manner. Aging itself is accompanied by increased accumulation of phosphorylated αSyn oligomers in monkeys^32^. Aging is associated with changes in the motor coordination^33^. Additionally, the neuroinflammatory process in Parkinson’s disease brain is possibly triggered by aging^34^. The PPARγ-knockout in mice does not cause the death of neuronal cells, but it can increase the susceptibility to brain damage after cerebral ischemia^35^. The small αSyn aggregates (oligomers and protofibrils) rather than the αSyn fibrils are reported to be toxic to neurons^14,36^. A progressive conversion of the soluble αSyn protein into insoluble, β-sheet-rich filaments, and their intraneuronal deposition into Lewy bodies underlie its cytotoxicity in the synucleinopathies^37^. The phosphorylated αSyn oligomers have a potential neurotoxic effect, although at the lower levels it may not induce obvious neurodegeneration. The accumulation of αSyn may increase the vulnerability of dopaminergic neurons to inflammation, as genetic ablation of αSyn decreases the sensitivity of cells to inflammatory challenges^38^. Taken together with the discussion, the seipin deficiency through reducing PPARγ causes the age-related loss of dopaminergic neurons probably by at least two possible mechanisms: one is increased oligomerization of αSyn, and the other is enhanced neuroinflammation and phosphorylation of αSyn via activation of GSK3β (Fig. 6).

## Conclusion

The seipin deficiency through enhanced aggregation and phosphorylation of αSyn or neuroinflammation causes the loss of dopaminergic neurons leading to deficits in motor coordination. Overall, the results presented herein can help for understanding underlying pathogenic processes and mechanisms of Parkinson’s disease.

## Materials and methods

### Experimental animals

All experiments were carried out in accordance with the guidelines established by the Institute for Laboratory Animal Research of Nanjing Medical University. All animals were housed in a light-controlled room under a 12-h light-dark cycle (starting at 07:00 h) and were maintained at 23 °C in the Animal Research Center of Nanjing Medical University. The animals had free access to food and water before and after all procedures. The *seipin*-sKO, *seipin*-nKO, and *seipin*-aKO mice were generated as previously described^11,19^. Briefly, neuron-specific and adipose-specific deletion of seipin exon 3 was induced by crossing mice with the loxP seipin allele with transgenic mice expressing Cre recombinase driven by a neuron-specific or an adipocyte-specific promoter (nestin-Cre or aP2-Cre; Jackson Laboratories). Progenies were screened by PCR for loss of the seipin exon 3 and presence of nestin-Cre or aP2-Cre. The genotype of *seipin*-nKO and *seipin*-aKO mice was identified by PCR using genomic DNA from their tails. When heterozygous seipin LoxP-nestin or aP2-Cre were obtained, they were further crossed with seipin loxP/loxP homozygous mice. The progenies were screened by genomic DNA PCR for seipin loxP/loxP with nestin-Cre or aP2-Cre, which would be used in subsequent experiments. Progenies were also screened by PCR for loss of the seipin exon 3 and presence of nestin-Cre, which would be used in subsequent experiments. The DNA was amplified with the following primers: 5′-CTTGTCTCAAAGGGGTCT-3′ (forward primer for loxP) and 5′-TCAACAGAACAGACGCT-3′ (reverse primer for loxP); and 5′-GCGGTCTGGCAGTAAAAACTATC-3′ (forward primer for nestin-Cre) and 5′-GTGAAACAGCATTGCTGTCACTT-3′ (reverse primer for nestin-Cre).

### Experimental design

Male 3-M-old (*n* = 22), 8-M-old (*n* = 66), and 12-M-old (*n* = 12) *seipin*-nKO mice, 8-M-old *seipin*-sKO mice (*n* = 12) or 8-M-old *seipin*-aKO mice (*n* = 12) and age-matched control mice (nestin-Cre mice) (*n* = 12) or WT mice (*n* = 62) were used at the beginning of all experiments and divided into five experimental groups: the first group was used to examine spontaneous activity and motor coordination, number of dopaminergic neurons, levels of αSyn monomers, αSyn oligomers, and oligomer phosphorylation; the second group was used to measure the levels of cleaved caspase-3, PPARγ expression, ER stressers, phosphorylation of GSK3β, IL-6, and TNF-α; the third group was examined the phosphorylation of αSyn in dopaminergic neurons; the fourth and fifth groups were treated with rosiglitazone or AR-A014418. At the end of the behavioral tests the mice were perfusion-fixed for histological examination or were decapitated for biochemical assays and western blotting.

### Behavioral examination

Three different behavioral tests were carried out (9000–1400) under the following sequence: open-field test → beam walking test → rotarod test. The tests were spaced by 24 h. These behavioral tests were recorded by a video monitor (Winfast PVR; Leadtek Research Inc., Fremont, CA). The results of the OFT and BWT were analyzed using TopScan Lite 2.0 (Clever Sys, Reston, VA), and the results of the RT were analyzed by Rota-Rod Microprocessor 47600 (Ugo Basile, Biological Research Apparatus, Varese, Italy). *Open-field test*: Each mouse was placed in a clear, open-top, square Plexiglas box (30 cm × 30 cm × 40 cm) in a subdued room and allowed to freely explore for 6 min. Total traveled distance was measured within 6 min. *Beam walking test*: The challenging beam was a 1-m long wooden beam suspended 23 cm above a bench top covered with soft pads. The beam was divided in four gradually narrowing sections (25 cm/section) at widths of 3.5, 2.5, 1.5, and 0.5 cm. The beam was covered with surgical tape that provided sufficient surface traction for the animals to walk on. All mice were pre-trained on traversing the beam for 2 consecutive days. On the third day, each mouse tested with five trials (inter-trial intervals = 10–12 s), and the average time was calculated^39^. *Rotarod test*: Each mouse was placed in the forward position on a rotating rod (rotarod apparatus, Ugo Basile, Varese, Italy). On days 1 and 2, the mice learned to stay on the rotarod at a constant speed (20 rpm) for 300 s. For a RT with constant speeds (RT-CS), motor coordination was assessed with five constant speeds (4–36 rpm) for a maximum of 60 s at each speed on day 3. For a RT with accelerating speed (RT-AS), motor coordination was assessed with an accelerating speed (4–45 rpm) over a period of 2.5 min. The mice were tested twice at each speed with a resting period of 20 min. For each trial, the time (latency) until the mice fall off the rotarod was recorded.

### Histological examination

Mice were anesthetized with chloral hydrate (400 mg/kg, i.p.) and then perfused with 4% paraformaldehyde. The brains were transferred into 30% sucrose. *TH immunohistochemistry and quantitative analyses*: coronal sections (30 μm) were cut using a cryostat after gradient dehydration. The sections were treated with 5% normal goat serum and then incubated in chicken anti-TH (1:1000; Abcam ab76442, Cambridge, UK) at 4 °C overnight. The sections were incubated in biotin-labeled goat anti-chicken IgG antibody (1:500; Abcam ab6876) for 2 h. Immunoreactivities were visualized by avidin-biotin horseradish peroxidase complex (ABC Elite; Vector Laboratories, Inc., Burlingame, CA, USA) using 3,3′-diaminobenzidine as a chromogen. The sections were observed using a microscope (Olympus DP70, Tokyo, Japan) with a 40× objective. Every third section (12 sections per mouse) was obtained for consecutive cell quantification analyses. Stereological cell counting was performed with a stereological system consisting of a light microscope with a CCD camera (Olympus DP70), a motorized specimen stage for automatic sampling (MicroBrightField, Williston, VT, USA) and a computer running MicroBrightField Stereo Investigator software (MicroBrightField)^40^. The section thickness was measured using a dissector height of 5 μm. The total number of TH-positive cells was estimated using the optical fractionator formula: number of neurons = 1/ssf (slice sampling fraction) × 1/asf (area sampling fraction) × 1/tsf (thickness sampling fraction) × Σ (number of objects counted). *TH/seipin or TH/p-αSyn dual-antigen immunofluorescence*: The sections (30 μm) were treated with 5% normal goat serum and then incubated in a chicken anti-TH antibody (1:1000; Abcam ab76442) and rabbit anti-seipin antibody (1:1000; Abcam ab106793)^41^ or rabbit anti-phosphor-αSyn antibody (1:1000; Abcam ab51253), at 4 °C overnight. The seipin monoclonal antibody was a kind gift from Professor Jiahao Sha (State Key Laboratory of Reproductive Medicine, Nanjing Medical University, China). The sections were incubated in Alexa Fluor 594-conjugated AffiniPure donkey anti-chicken antibody (1:200, Jackson ImmumoResearch, West Grove, PA), Alexa Fluor 488-conjugated Affinipure donkey anti-rabbit antibody (1:200, Jackson ImmumoResearch) for 2 h. Sections were coverslipped with Antifade Mounting Medium (Sigma-Aldrich) and viewed under a fluorescent microscope (Olympus DP70) with a 100× objective. *Double immuno-staining of TH/Thioflavin S*: The αSyn fibril formation was monitored by Th-S binding. Th-S is a fluorescent dye that interacts with fibrils in a β-sheet structure. The sections (30 μm) were pre-incubated with 5% normal horse serum and then incubated in a chicken anti-TH antibody at 4 °C overnight. After phosphate-buffered saline (PBS) rinses, the sections were incubated Alexa Fluor 594-conjugated AffiniPure donkey anti-chicken antibody (1:200, Jackson ImmumoResearch) for 2 h. Then, the sections were incubated in Thioflavin S (10 µg/ml; Sigma-Aldrich) for 5 min. After PBS washing, sections were coverslipped with Antifade Mounting Medium (Sigma-Aldrich) and viewed using a fluorescent microscope (Olympus DP70) with a 100× objective.

### Western blot analysis

The brains were removed quickly under anesthesia with isoflurane. The coronal sections (500 μm) of midbrain were cut using a cryostat. The regions of containing SN were harvested using a 15-gauge needle (inner diameter = 1.5 mm). The samples were homogenized in lysis buffer (50 mM Tris-HCl, 150 mM NaCl, 5 mM EDTA, 10 mM NaF, 1 mM sodium orthovanadate, 1% TritonX-100, 0.5% sodium deoxycholate, 1 mM phenylmethylsulfonyl fluoride, and protease inhibitor cocktail) (Roche, Germany). The protein concentration was measured using a BCA protein assay kit (Pierce, IL, USA). For SDS-PAGE experiments, proteins were suspended in non-reducing loading buffer (0.3 M Tris-HCl, 5% SDS, and 50% glycerol) or reducing loading buffer (0.3 M Tris-HCl, 5% SDS, 50% glycerol, and 100 mM dithiothreitol) (Pierce), and then boiled. For native-PAGE experiment, the samples were homogenized in solubilization buffer containing 50 mM BisTris-HCl (pH 7.0), 500 mM 6-amino-caproic acid, 10% glycerol, 1% digitonin (20% stock in water), cocktail and phosphatase inhibitor, and then suspended in loading buffer (5% Serva G250, 50 mM BisTris-HCl, 500 mM 6-amino-caproic acid) without boiling directly onto gels^42^.

After transfer of proteins to polyvinylidene difluoride membranes (Millipore), the membranes were incubated with 5% nonfat dried milk for 60 min and then incubated with rabbit anti-seipin (1:1000; Abcam ab106793), rabbit anti-phosphorylated αSyn (1:1000; Abcam ab51253), rabbit anti-phosphorylated GSK3β (S9, 1:1000; Cell Signaling Technology 9336S, Inc., Boston, MA, USA), mouse anti-phosphorylated GSK3β (Y216, 1:1000; BD Transduction Laboratories 612313, Lexington, KY, USA), mouse anti-cleaved caspase-3 (c-caspase-3, 1:1000; Cell Signaling Technology 9661S), rabbit anti-PPARγ (1:1000; Santa Cruz sc-7196, Fremont, CA, USA), rabbit anti-GRP78 (1:1000; Bioworld Technology BS1154, St. Louis Park, MN, USA), or rabbit anti-CHOP (1:500; Cell Signaling Technology 2895S) at 4 °C overnight. The membranes were incubated with horseradish peroxidase-labeled secondary antibodies and visualized using an ECL detection kit (Millipore), and images were acquired using the ChmiDOC XRS Imaging System (Bio-Rad Laboratories, CA, USA). Optical density of the immunoreactive bands was measured using NIH ImageJ Software (Bethesda, MD, USA). Following visualization, the blots were stripped by incubation in stripping buffer (Restore, Pierce) for 5 min, re-blocked with 5% nonfat dried milk for 60 min, and re-incubated with rabbit anti-GSK3β (1:1000; Cell Signaling Technology 9315S), rabbit anti-caspase-3 (1:1000; Cell Signaling Technology 9662S), or mouse anti-β-actin (1:1000; Abbkine A01010, Redlands, CA, USA) or mouse anti-αSyn (anti-synuclein-1, 1:1000; BD Transduction Laboratories 610786, Lexington, KY, USA). The optical density of specific bands was then normalized to the corresponding GSK3β, αSyn, or β-actin levels.

### Treatment with drugs

The PPARγ agonist rosiglitazone (rosi; Enzo, Farmingdale, NY, USA) and the GSK3β antagonist AR-A014418 (AR; Sigma-Aldrich, MO, USA) were dissolved in dimethyl sulfoxide (DMSO) and then diluted in saline to a final concentration of 0.5% DMSO. Oral administration (p.o.) of rosi (5 mg/kg)^43^ or intraperitoneal injection (i.p.) of AR-A014418 (1 mg/kg)^44^ was given daily.

### Enzyme-linked immunosorbent assay (ELISA)

The samples of SN were homogenized in PBS solution and centrifuged at 12,000 rpm for 15 min at 4 °C, and the supernatant was obtained for further analyses. Sample aliquots of 100 μl were used to measure TNF-α and IL-6 using mouse cytokine ELISA kits from R&D Systems (Minneapolis, MN), according to the manufacturer’s instructions. The absorbance for all of the cytokines studied was measured using a SpectraMax Plus 384 Microplate Reader (Molecular Devices, MD, USA) at 450 nm.

### Quantitative real-time reverse-transcription PCR

The samples of the SN were quickly taken, and total RNA was isolated using TRIzol reagent (Invitrogen, Carlsbad, CA, USA). RNA (1 μg) was used to reverse transcribe using high-capacity cDNA of the reverse transcription kit RT (TaKaRa Biotechnology Co., Ltd.). The primer sequences of *PPARγ* (forward primer: 5′-GCTTATTTATGATAGGTGTGATC-3′; reverse primer: 5′-GCATTGTGAGACATCCCCAC-3′) and *GAPDH* mRNA (forward primer: 5′-ACCACAGTCCATGCCATCAC-3′; reverse primer: 5′-ACCACAGTCCATGCCATCAC-3′) were designed according to a previous publication^11^. Q-RT-PCR was performed using a Light Cycler Fast Start DNA Master SYBR Green I kit and an ABI Prism 7300 Sequence Detection System (Applied Biosystems, Foster City, California, USA). The relative expression of genes was determined using the 2-ΔΔct method, with normalization to *GAPDH* expression. The levels of *PPARγ* mRNA were expressed as percent of control mice.

### Data analysis/statistics

Data were retrieved and processed with Microcal Origin 8.0. The group data were expressed as the mean ± standard error. All statistical analyses were performed using SPSS software, version 20.0 (SPSS Inc., USA). Differences among the means were analyzed using the analysis of variance (ANOVA) with or without repeated measures, followed by post hoc Bonferroni’s test, where appropriate. Differences at *P* < 0.05 were considered to be statistically significant.
